# The therapeutic role of naringenin nanoparticles on hepatocellular carcinoma

**DOI:** 10.1186/s40360-024-00823-w

**Published:** 2025-01-03

**Authors:** Aya G. Elwan, Tarek M. Mohamed, Doha M. Beltagy, Doaa M. El Gamal

**Affiliations:** 1 Biochemistry Department, Faculty of Science, Tanta University, Tanta, Egypt; 2 Biochemistry Department, Faculty of Science, Damanhour University, Damanhour, Egypt

**Keywords:** Cancer, Hepatocellular carcinoma, Naringenin, Naringenin nanoparticles

## Abstract

**Background:**

Naringenin, a flavonoid compound found in citrus fruits, possesses valuable anticancer properties. However, its potential application in cancer treatment is limited by poor bioavailability and pharmacokinetics at tumor sites. To address this, Naringenin nanoparticles (NARNPs) were prepared using the emulsion diffusion technique and their anticancer effects were investigated in HepG2 cells.

**Methods:**

The particle size of NARNPs was determined by transmission electron microscopy and scanning electron microscopy analysis. NARNP is characterized by Fourier transform infrared spectroscopy and X-ray diffraction. Study the cytotoxic effects of various doses of naringenin, NARNPs and DOX on HepG2 and WI38 cell lines after 24 h and 48 h using the MTT assay. Flow cytometric analysis was used to study the apoptotic cells. The study also examined the expression of apoptotic proteins (p53) and autophagy-related genes ATG5, LC3 after treatment with naringenin, NARNPs, doxorubicin, and their combinations in HepG2 cells.

**Results:**

The particle size of NARNPs was determined by transmission electron microscopy and scanning electron microscopy analysis, showing mean diameters of 54.96 ± 18.6 nm and 31.79 ± 6.8 nm, respectively. Fourier transform infrared spectroscopy confirmed successful conjugation between naringenin and NARNPs. NARNPs were in an amorphous state that was determined by X-ray diffraction. The IC50 values were determined as 22.32 µg/ml for naringenin, 1.6 µg/ml for NARNPs and 0.46 µg/ml for doxorubicin. Flow cytometric analysis showed that NARNPs induced late apoptosis in 56.1% of HepG2 cells and had no cytotoxic effect on WI38 cells with 97% viable cells after 48 h of incubation. NARNPs induced cell cycle arrest in the Go/G1 and G2/M phases in HepG2 cells. The results showed increased expression of ATG5, LC3, and p53 in HepG2 cells treated with IC50 concentrations after 48 h of incubation. NARNPs enhanced the cytotoxic effect of doxorubicin in HepG2 cells but decreased the cytotoxic effect of doxorubicin in WI38 cells.

**Conclusions:**

The study demonstrated that NARNPs effectively inhibit cell proliferation and induce apoptosis in human hepatocellular carcinoma cells. Importantly, NARNPs showed no cytotoxic effects on normal cells, indicating their potential as a promising therapy for hepatocarcinogenesis. Combining NARNPs with chemotherapy drugs could present a novel approach for treating human cancers.

## Introduction

Cancer is a cellular malignancy characterized by the abnormal growth of cells resulting from disruptions in the cell cycle. This uncontrolled growth leads to the formation of tumors that can invade surrounding tissues and spread to other parts of the body [1, 2]. This complex biological disease is influenced by various environmental and hereditary factors, including radiation, smoking, ultraviolet rays, stress, and pollution. These factors can induce cellular changes such as impaired apoptosis, oxidative stress, and genetic mutations [3, 4]. The challenges of anticancer treatments are often attributed to cancer cells’ ability to resist medication, leading to severe side effects associated with surgeries, radiation, and chemotherapy, especially in advanced stages of cancer [4]. To mitigate the adverse effects of cancer therapy drugs on healthy organs, it is crucial to develop therapeutic approaches that minimize such impacts [5].

The World Health Organization (WHO) ranks liver cancer as the third leading cause of cancer-related mortality and as the fifth leading cause of incident cases worldwide [6]. Hepatocellular carcinoma (HCC) constitutes approximately 90% of all primary liver cancer cases [7]. Malignant transformation of hepatocytes is the usual cause of the complex pathophysiological mechanism of HCC [8]. Due to the absence of specific symptoms in the early stages, most patients are diagnosed at an advanced stage, leading to a poor prognosis [9]. Hepatocellular carcinoma (HCC) is a complex disease that often arises from cirrhosis. Managing HCC requires a multidisciplinary approach involving diagnosis, staging, treatment, and monitoring in specialized outpatient clinics to slow the disease’s progression. Although significant advances have been made in the clinical treatment of HCC over the past decade, the disease still has a high rate of recurrence even after ablation or surgical resection [10]. Surgical resection, liver transplantation, percutaneous local ablation, transarterial embolization, chemotherapy, and radiation therapy are among the options for treating HCC. Additionally, it is critical that cancer treatments destroy cancer cells without damaging healthy cells, which is particularly critical for patients with advanced cancer. Hepatocellular cells have a number of complex strategies for avoiding immune recognition, which prevents them from programmed death [11]. Thus, one of the main objectives of anticancer treatments is to induce apoptosis. It has recently been demonstrated that a number of naturally occurring substances can induce apoptosis in cancer cells. These drugs may also be combined with chemotherapy or radiation therapy, which can improve the therapeutic efficacy and reduce the adverse effects of many cancer treatments [12, 13]. Because of their strong anticancer activity and minimal side effects, natural products are therefore regarded as potential candidates [14].

In recent years, researchers have shown growing interest in utilizing dietary phytochemicals for the treatment of various malignancies [15]. Flavonoids, a group of natural compounds, exhibit pharmacological properties including antioxidant and anti-carcinogenic effects [16–19]. Dietary phytochemicals have been shown to inhibit cell proliferation, induce apoptosis, and promote autophagy, highlighting their significant potential for cancer prevention [20–22]. Many anti-cancer therapies, used for both solid and blood-related cancers, can lead to vascular and metabolic complications such as hypertension, atherosclerosis, coronary artery disease, and thromboembolic events [23]. Vascular disorders, particularly endothelial dysfunction, are crucial in initiating and worsening cardiac and renal diseases. Adenosine, a purine nucleoside released during metabolic stress or cellular damage, plays a protective role by regulating vascular responses and facilitating adaptation to stress conditions [24, 25]. Flavonoids modulate vascular and metabolic pathways by targeting specific enzymes like adenosine deaminase and cyclic nucleotide phosphodiesterases. They also interact with adenosine receptors, highlighting their potential role in regulating vascular function and stress responses [26]. Plant bioflavonoids such as naringenin (40, 5, 7-trihydroxyflavonone) are present in citrus, grapefruit, and tomato fruits. Pharmacological evaluations of naringenin’s potential anticancer, anti-mutagenic, antiatherogenic, hepatoprotective, anti-inflammatory, and nephroprotective properties have already been conducted [27]. Several in-vitro studies have demonstrated that naringenin exhibits growth-inhibitory and cytotoxic effects across a variety of cancer cell lines, including breast (MCF-7, MDA-MB-231), stomach (KATOIII, MKN-7), liver (HepG2, Hep3B, Huh7), cervical (HeLa, HeLa-TG), pancreatic (PK-1), colon (Caco-2), and leukemia (HL-60, NALM-6, Jurkat, U937) [28]. In human lung cancer A549 cells, it enhanced TRAIL-mediated apoptosis [29]. Naringenin also increased apoptosis in AGS gastric cancer cells by inhibiting the β-catenin/Tcf signaling pathway, which is critical in cancer development [30]. Additionally, in human promyelocytic leukemia HL-60 cells, naringenin promoted cell death by activating NF-kB, leading to ATP depletion and mitochondrial dysfunction [31]. Naringenin’s ability to suppress colorectal cancer is linked to its inhibition of COX-1, a key enzyme in cancer progression [32]. Its protective effects against DNA damage and inhibition of cancer cell proliferation may stem from its capacity to block the synthesis of polyamines—molecules essential for the regulation of protein, DNA, and RNA synthesis. Furthermore, it disrupts DNA-protein interactions, which are crucial for maintaining normal cellular functions [33, 34].

Despite its promising anticancer potential, the application of naringenin in cancer treatment is hindered by its limited bioavailability at tumor sites and suboptimal pharmacokinetics. Naringenin possesses three hydroxyl groups in its aromatic rings, contributing to its strong antioxidant and pharmacological properties [35]. Naringenin is a promising therapeutic agent with potential applications for treating a variety of diseases. However, its use in treating acute liver injury is limited by challenges such as insufficient liver retention, low bioavailability, and poor water solubility. Therefore, it is crucial to enhance naringenin’s solubility, bioavailability, and liver targeting. The rapid advancement of nanotechnology presents a practical solution to improving a drug’s water solubility, enhancing its bioavailability, reducing instability and poor permeability, and targeting specific organs such as the liver [36–38]. Polymeric nanoparticles have been used to increase naringenin’s bioavailability and efficacy [39, 40]. Due to their small size and numerous advantages, such as enhanced cellular and tissue targeting, targeted effects in particular tissues, and increased oral bioavailability, these nanoparticles have attracted attention [41, 42].

Therefore, the objective of the current study was to develop naringenin nanoparticles (NARNPs), evaluate their anticancer effectiveness in the HepG2 cell line, and explore their potential protective effects against chemotherapy-induced side effects in hepatocellular carcinoma.

## Materials and methods

### Chemicals

All chemicals used in this study were purchased from Sigma Aldrich (St. Louis, MO, U.S.A.). Naringenin (CAS-No. 480-41-1), Polyvinyl alcohol (PVA)(CAS-No.9002-89-5),3-(4,5-dimethylthiazoil2-yl)-2,5 diphenyltetrazolium bromide(MTT) from Sigma–Aldrich, USA (CAS-No. 298-93-1), Dulbeccos’ Modified Eagle medium (DMEM) (Cambrex bioscience, Verviers, Belgium), RPMI-1640 with L-glutamine (Cambrex bioscience, Verviers, Belgium), Fetal bovine serum (Cambrex bioscience, Verviers, Belgium), Penicillin-streptomycin solution (Cambrex bioscience, Verviers, Belgium), FITC Annexin V apoptosis detection kit from Immunostep Co (Spain), RNA extraction kit (Thermo Scientific, Fermentas, K0731), Reverse transcription kits (Thermo Scientific, Fermentas, EP0451), SYBR Green/ROX qPCR Master Mix (Thermo Scientific, USA, K0221), and gene-specific primers.

### Preparation of naringenin nanoparticles

Naringenin nanoparticles (NARNPs) were prepared using the emulsion diffusion method in a 1:1 ratio of Naringenin to PVA [43]. To achieve this, 10 ml of ethanol was used to dissolve 1 g of naringenin, and 1 g of PVA was dissolved in 10 ml of water. The two mixtures were then heated for 30 min to complete the dissolution process. The PVA solution was added drop by drop to the naringenin solution and stirred with a hot plate for 30 min and then centrifuge at 2000 rpm for 30 min. Finally, the mixture was lyophilized using a freeze dryer.

### Characterization of nanoparticles

#### Transmission electron microscopy (TEM) analysis

The size of NARNPs is determined by JEM-1400 (Japan). A suspension of 0.5 mg of NARNPs in 1 ml of water was sonicated for 30 s. One drop of the suspension nanoparticle was applied to the copper grid and stained for 10 min with 1% uranyl acetate before drying and visualized the image at 100 kV.

#### Scanning electron microscopy (SEM) analysis

The morphology of NARNPs was analyzed by scanning electron microscopy (JEOL SEM, JSM-636OLA, Japan) at an accelerated voltage of 20 KV.

#### Fourier transform infrared spectroscopy (FT-IR)

Naringenin and NARNPs were analyzed by a Perkin Elmer, USA FT-IR spectrometer. A hydraulic press was used to compress 5 mg of sample into a pellet after mixing it with 100 mg of KBr. The range of FT-IR spectra was 4000–400 cm − 1.

#### X-ray diffraction (XRD) analysis

Naringenin and NARNPs were analyzed at wavelength 0.1546 nm by using X-ray at a voltage of 40 kV and 30 mA.

### Cell culture

Two types of cell lines were used for in vitro studies: the human hepatocellular carcinoma HepG2 cell line (cancerous cells) and the human diploid lung fibroblast cell line WI38 (normal cells). These cell lines were obtained from the Serum and Vaccine institution, Cairo. Cells were cultured in RPMI and DMEM media with 10% fetal bovine serum and a Penicillin-streptomycin solution in a humidified 5% CO2 incubator. The study involved five groups: control group, naringenin group, NARNPs group, DOX group, and DOX combined with NARNPs group. Cell viability was assessed using the MTT assay, a non-radioactive calorimetric method for determining cell cytotoxicity in vitro [44, 45].

### Cell viability assay

HepG2 and WI38 were cultured in 96-well plates at a density of 10 × 10^3^ per well and cultured with media for 24 and 48 h. After incubation, the cells were treated with different concentrations of naringenin (6.8, 13.5, 27.2, 54.4, 108.9, 272.2 µg/ml), NARNPs (2,4,8,10,20,50 µg/ml ), DOX (0.2, 0.4, 0.8, 2, 4,8 µg/ml), control cells were untreated and incubated for 24 and 48 h at 37 °C in 5% CO2. After the treatment period, the medium was withdrawn, and new media containing MTT solution (1 mg/mL) was added. The cells were then incubated for an additional 4 h at 37 °C. The amount of formazan produced was dissolved in 100 µL of DMSO and measured using a spectrophotometer at 570 nm. Prism software was used to determine the IC50 values for naringenin, NARNPs, and DOX [44]. Based on the IC50 results from the MTT assay, DOX was combined with NARNPs in the following ratios:

- 1:1( 50 µl of 0.46 µg/ml DOX: 50 µl of 1.6 µg/ml NARNPs),
- 1:2 ( 33.3 µl of 0.46 µg/ml DOX: 66.7 µl of 1.6 µg/ml NARNPs),
- 1:3 (25 µl of 0.46 µg/ml DOX: 75 µl of 1.6 µg/ml NARNPs).

### Annexin V assay

Annexin V-fluorescein isothiocyanate and PI staining protocol was followed for HepG2 and WI38 cells [46]. Flow cytometric analysis was used to study the apoptotic cells. A total of 2 × 105 HepG 2 and WI38 cells were plated in 6-well plates and subjected to 22.32 µg/ml of naringenin, 1.6 µg/ml of NARNPs, 0.46 µg/ml of DOX, and DOX combined with NARNPs in a ratio of 1:3 for 48 h. The cells were stained using a propidium iodide and Annexin V-fluorescein isothiocyanate double-stained kit according to the manufacturer’s instructions, and flow cytometry was used to analyze the results [47–49].

### Analysis of cell cycle

After 48 h of treatment with the IC50 concentration of each drug, HepG2 and WI38 cells were centrifuged for 5 min at 1000 rpm after being fixed in 70% ethanol at 4 °C overnight. The pellets were then twice-washed in PBS. Then, after 30 min of incubation at 37 °C with 50 µg/ml propidium iodide and 100 µg/ml RNase A, cell pellets were resuspended in 0.5 ml of PBS and examined using a flow cytometer. Each analysis required the use of at least 10,000 cells, and the results were displayed as histograms.

### Reverse transcription-PCR

HepG2 and WI38 cells were cultured in 6 well plates with a density of 1 × 10^6^ cells/mL for 48 h in an incubator. After incubation, these plates were treated with IC50 concentrations for evaluation of the effect of these drugs on the mRNA expression of the autophagic and apoptotic genes. Collect culture cells from 6 well plates and extracted RNA according to kit instructions (Thermo Fisher Scientific, Waltham, MA, USA, Gene JET RNA extraction kit (Cat#K0731). Quantify the concentration of RNA extracted by Nano-drop spectrophotometer (Uv-Vis spectrophotometer Q5000/USA). Reverse transcription of mRNA was performed by (SensiFAST™ cDNA Synthesis Kit Thermo Co, BIO-6505, USA). In a nuclease-free PCR tube, 5 µg of template RNA were added, 4 µl of 5x Reaction Buffer. One µl of reverse transcriptase enzyme was added to the mixture then the volume was completed to 12.5 µl using DEPC-treated water.

Following the manufacturer’s instructions, the extracted cDNA was amplified using 2X Maxima SYBR Green/ROX qPCR Master Mix and gene-specific primers (Table 1). The polymerase chain reaction mixture was carried out in a 25 µl. 3 µl cDNA template (10–20 ng/ µl), 12.5 µl Maxima SYBR Green/ROX qPCR Master Mix, 1 µl Primer forward (10 µM), 1 µl Primer reverse (0.1–0.5 µM), 7.5 µl Water, nuclease free. Tubes were placed in the machine and subjected to a cycling program. Taq polymerase was used to perform PCR in 45 cycles (20 s at 95 °C, 15 s at annealing temperature at 57 °C and 30 s at 72 °C). Initial denaturation at 95 °C for 15 min.

**Table 1:** Primer sequence used for RT-PCR

Gene	Primer sequence
LC3	F = TTCCGAGTTGCTGACTGACC
	R = CCCTTGTAGCGCTCGATGA
ATG5	F = GCAACTCTGGATGGGATTGC
	R = TTGCAGCAGCGAAGTGTTTC
P53	F = CCTTCCCAGAAAACCTACCA
	R = TCATAGGGCACCACCACACT
GAPDH	F = TGCACCACCAACTGCTTAGC
	R = GGCATGGACTGTGGTCATGAG

All data were analyzed using one-way analysis of variance (ANOVA), followed by Dunnett’s test for significance between the different groups, and the results are presented as mean ± SD (*** *P* < 0.0001 for significant with control, n.s. for non-significant with control group).

## Results

### Characterization of naringenin nanoparticles (NARNPs)

#### TEM and SEM analysis

**Fig. 1:**
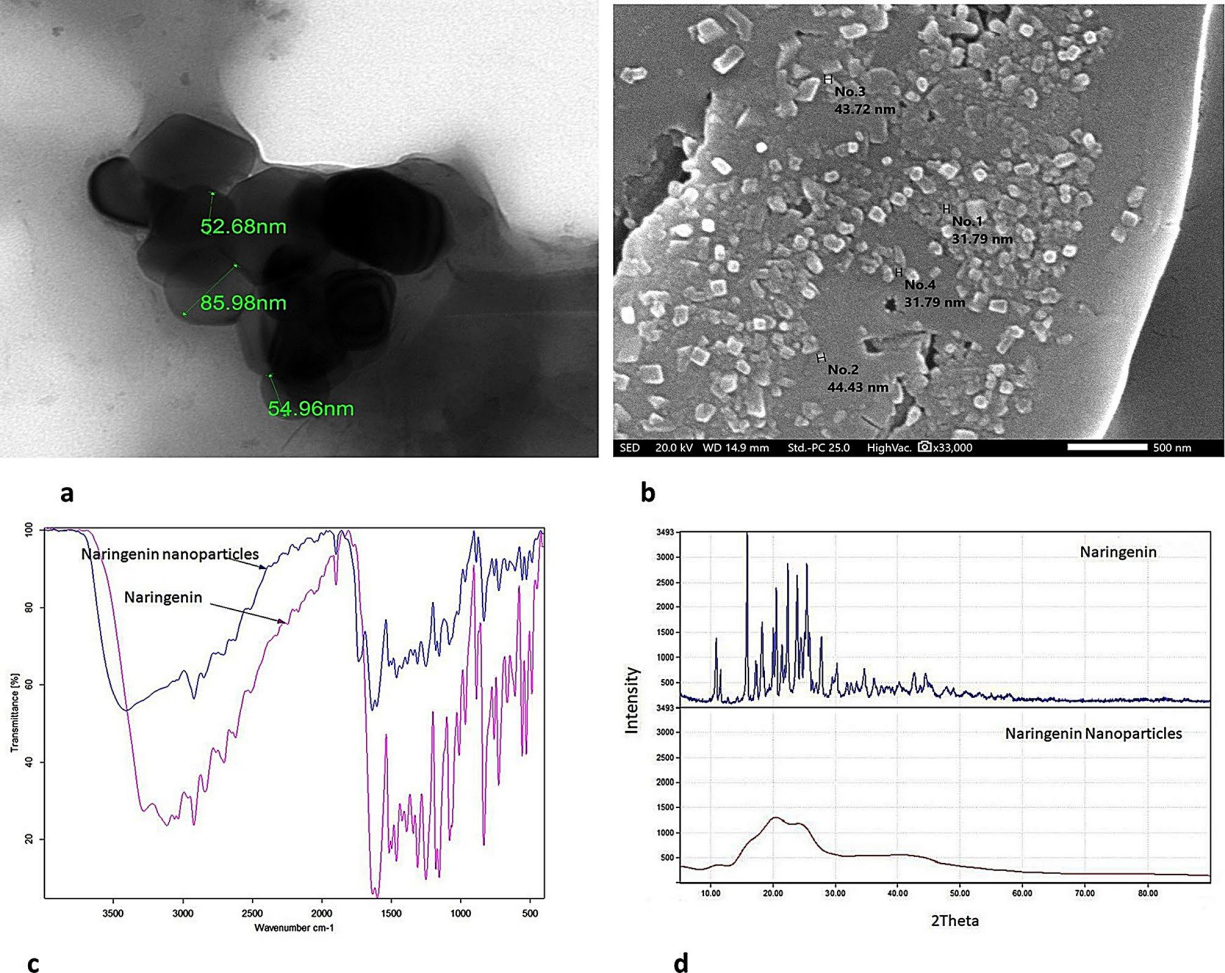
**a** Transmission electron microscopic (TEM) of NARNPs with average size 54.96 ± 18.6 nm. **b** Scanning electron microscopic (SEM) of NARNPs with average size 31.79 ± 6.8 nm. **c** The FT-IR spectra for naringenin and NARNPs. **d** X-ray diffraction (XRD) patterns for Naringenin and NARNPs

The particle size of NARNPs was determined using TEM and SEM analysis, showing mean diameters of 54.96 ± 18.6 nm and 31.79 ± 6.8 nm, respectively (Fig. 1a, b).

#### FT-IR spectroscopy

The chemical stability of the nanoparticle drug is measured by FT-IR spectroscopy (Fig. 1c). The existence of distinctive peaks at 2883 due to CH2 symmetric stretching, 1725 cm^− 1^ due to C O carbonyl stretching, and 1650 cm^− 1^ due to -CONH amide band in NARNPs, FT-IR spectra verified the successful conjugation between naringenin and NARNPs [50]. Due to the existence of various functional groups, free naringenin displayed distinctive bands. The peak shown at 2907 cm^− 1^ is caused by CH2 asymmetric stretching vibrations, whereas a band emerging at 3262 cm^− 1^ is caused by N-H/O-H stretching vibrations. The -CONH amide is responsible for the bands at 1654 and 1464 cm^− 1^. C-O stretching and C-O-C stretching vibrations could be the cause of the infrared bands at 1106 and 834 cm^− 1^, respectively. These distinct free-Naringenin bands are also present in NARNPs, demonstrating the chemical stability of the formulation of these nanoparticles [38, 51].

### X-ray diffraction analysis

Naringenin exhibited distinct peaks at 20.1° in its XRD spectrum, with several peaks in the 10–30° range. The crystalline peak numbers for naringenin are 10.79, 11.45, 15.85, 16.51, 17.25, 18.15, 18.6, 20.4, 21.32, 22.28, 23.81, 24.46, 25.37, 25.99, 26.35, and 27.71 (Fig. 1d). Naringenin’s characteristic peak disappeared from the NARNPs spectrum, suggesting that the substance is in an amorphous state and that the crystal structure was destroyed during the nanoparticle preparation process [38, 52].

#### Cytotoxic effect of naringenin, NARNPs on HepG2 cell line by using MTT assay

**Fig. 2:**
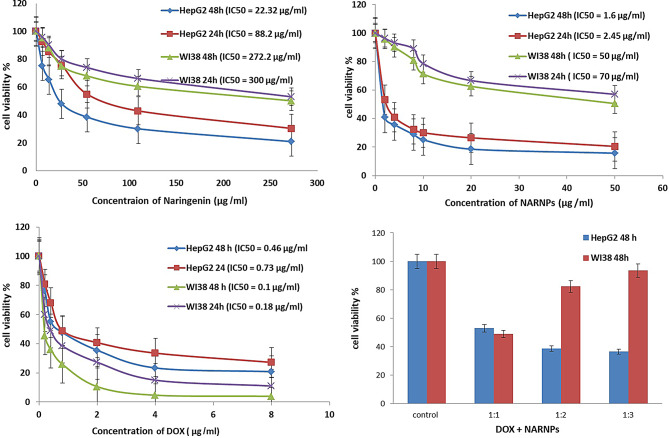
Cytotoxic activity of Naringenin, NARNPs, DOX and DOX combined with NARNPs on HepG2and WI38 cells after 48 h and 24 h incubation. Cell viability was measured using MTT assays. Cells were treated with different concentrations of naringenin (6.8–272.2 µg/ml), NARNPs (2–50 µg/ml) and DOX (0.2–8 µg/ml). A two way Anova test was carried out ***P < 0.0001 for significant with control

Study the cytotoxic effects of various doses of naringenin, NARNPs and DOX on HepG2 and WI38 cell lines after 24 h and 48 h using the MTT assay. Naringenin treatment significantly decreased the growth of cells in doses (6.5–272.2 µg/ml), NARNPs in dose (2–50 µg/ml) and Dox (0.2–8 µg/ml) after 24 h and 48 h of incubation. The IC50 was 22.32 µg/ml for naringenin, 1.6 µg/ml for NARNPs, 0.46 µg/ml of DOX and combination between DOX and NARNPs (1:3) (Fig. 2).

### Apoptosis analysis by flow cytometry

#### Annexin v assay

**Fig. 3:**
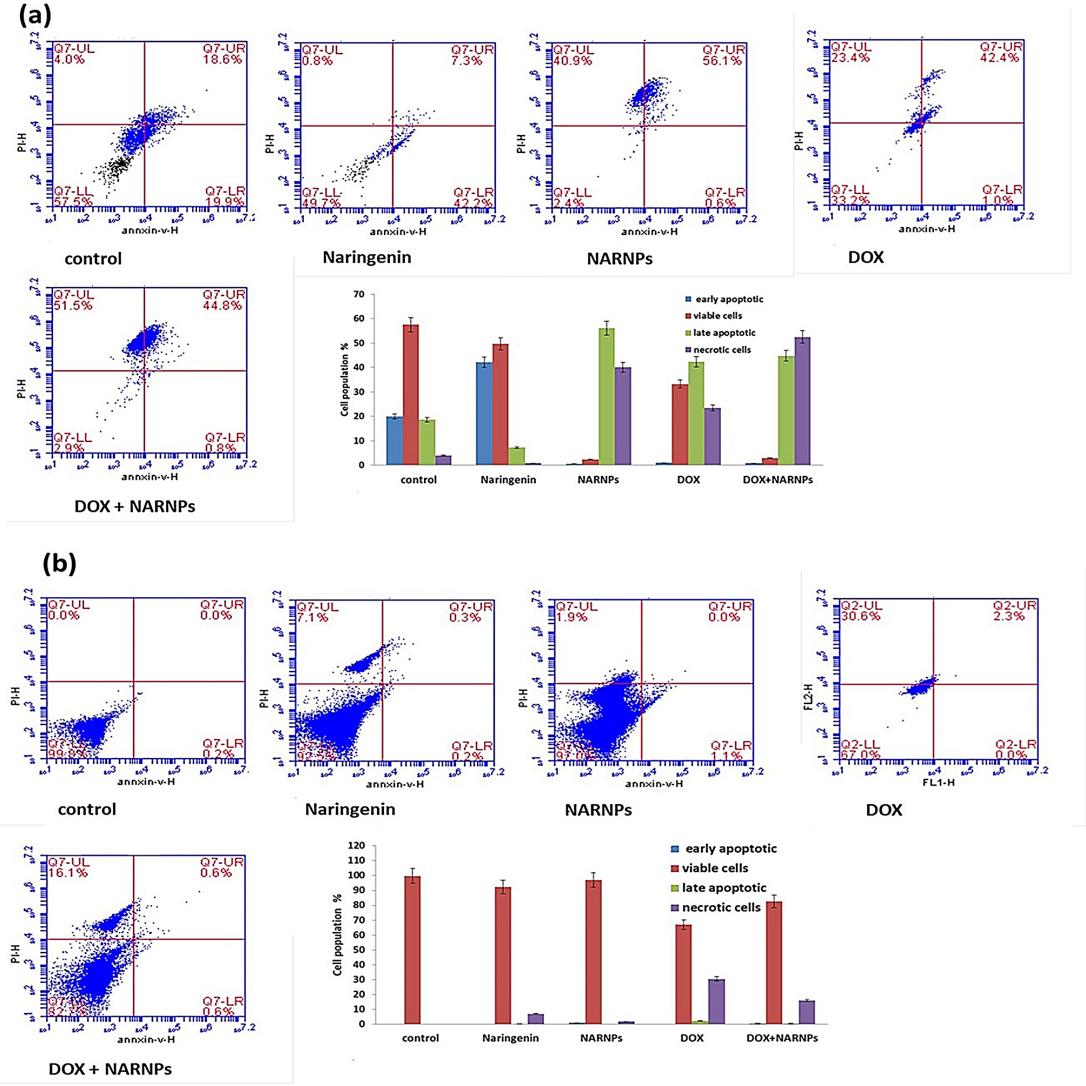
Flow cytometric analysis of **a** HepG2 cell line, **b** WI38 cell line incubated for 48 h. Plots shows Annexin V-FITC and propidium iodide stained. Cells in the lower left quadrant are alive, cells in the lower right quadrant are in early apoptosis, in the upper right are in late apoptosis, and cells in the upper left quadrant are necrotic cell. A two way Anova test was carried out *** *P* < 0.0001 for significant with control

The apoptotic cells attach to annexin V on phosphatidyl serine (PS) residues on their extracellular membrane. Apoptosis induction was assessed using Annexin V-FITC/PI staining. In untreated control, HepG2 cells, the percentages of late apoptotic, necrotic, viable, early apoptotic cells were 18.6%, 4%, 57.5, 19.9% respectively. Incubation of HepG2 for 48 h with IC50 of NARNPs followed by flow cytometric analysis resulted in 56.1% of cells being in late apoptotic, 40.9% in necrotic, 2.4% viable and 0.6% early apoptotic cells. The incubation of HepG2 with IC50 of naringenin resulted in 42.2% in early apoptosis. Incubation of HepG2 for 48 h with IC50 of DOX results in 42.4% of cells in late apoptotic, 23.4% of cells in necrotic, 1% in early apoptotic and 33.2% in viable cells. Incubation of HepG2 cells for 48 h with the IC50 of DOX combined with 1.6 µg/ml of NARNPs in a 1:3 ratio resulted in 44.8% of cells being in late apoptotic, 51.5% in necrotic, 0.8% in early apoptotic and 2.9% in viable cells. In contrast, incubation of WI38 cells for 48 h with the IC50 of NARNPs and naringenin resulted in 97% and 92% viable cells, respectively, indicating minimal cytotoxic effects on normal cells, 1.1% and 0.2% early apoptotic cell, respectively, 0% and 0.3% in late apoptotic cell, respectively and 1.9% and 7.1% in necrotic cells. Effect of DOX on WI38 result in 30.6% necrotic cells, 67% viable cell, 2.3% late apoptotic cells, so doxorubicin has cytotoxic effect on normal cell. Combination of NARNPs with DOX decrease the cytotoxic effect of doxorubicin on normal cell and result in 82.7% viable cells, 0.6% late apoptotic, 0.6% early apoptotic and 16.1% in necrotic cells, so the combination of NARNPs with DOX decrease the cytotoxic effect of DOX on normal cells (Fig. 3a, b).

### Effect of NARNPS and naringenin on the cell cycle distribution

**Fig. 4:**
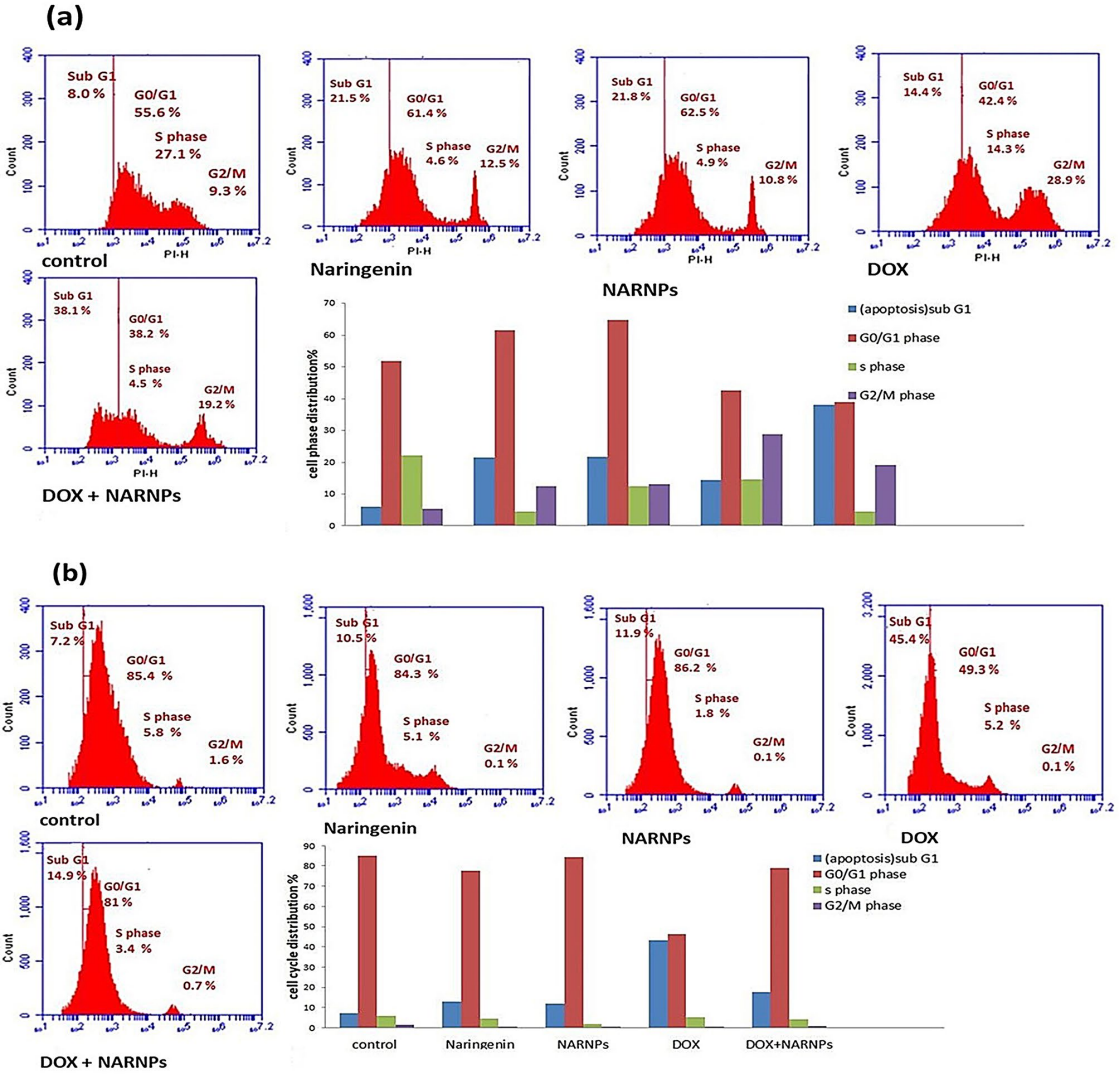
Cell cycle analysis of **a** HepG2 cell line, **b** WI38 cell line incubated for 48 h. A two way Anova test was carried out *** *P* < 0.0001 for significant with control

NARNPs and naringenin induced HepG2 cell cycle arrest at the Go/G1 and G2/M phase. Exposure to 22.32 µg/ml of naringenin increased cells in the Go/G1 phase from 55.6 to 61.4%, in the G2/M phase increased from 9.3 to 12.5%, and in Sub G1 phase increased from 8 to 21.5%. While exposure to 1.6 µg/ml of NARNPs increased cells in the Go/G1 phase from 55.6 to 62.5%, in the G2/M phase increased from 9.3 to 10.8% and in the Sub G1 phase increased from 8 to 21.8%. Exposure to 0.46 µg/ml DOX the percentage of Hep G2 cells at the Go/G1 phase decreased from 55.6 to 42.4%, and in G2/M phase increased from 9.3 to 28.9%. Combining DOX with NARNPs (0.46–1.6 µg/ml) decreased cells in the Go/G1 phase from 55.6 to 38.2% and increased cells in the G2/M phase from 9.3 to 19.2% (Fig. 4a, b).

#### Effect of naringenin, NARNPs, DOX and DOX combined with NARNPs on the expressions of apoptotic and autophagic proteins

**Fig. 5:**
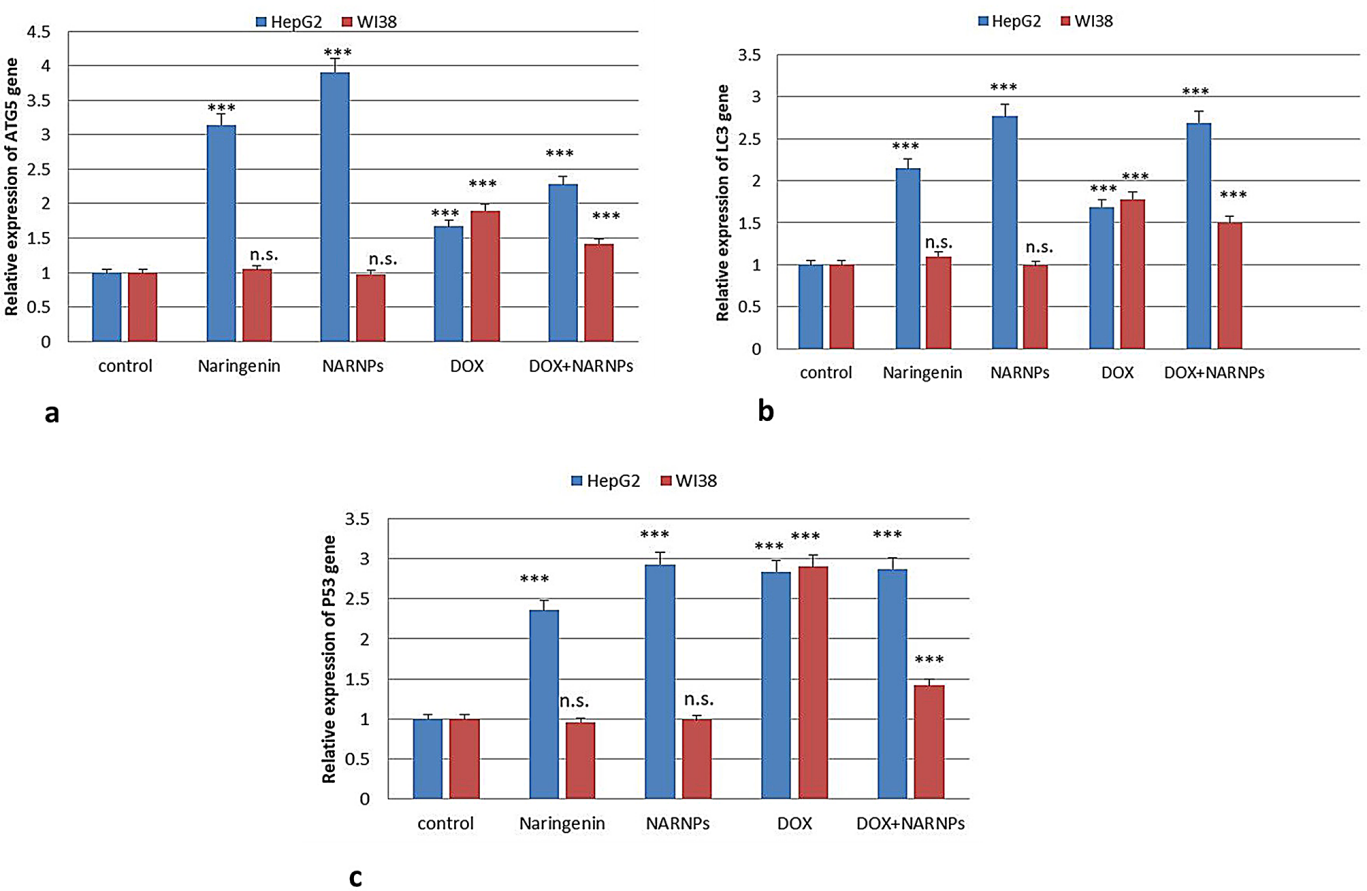
Real-time PCR analysis of ATG5, LC3 and P53 mRNA expression in HepG2 and WI38 cells incubated 48 h with naringenin, NARNPs, DOX and DOX combined with NARNPs. A two way Anova test was carried out ***P < 0.0001 for significant with control, n.s. for non-significant with control group

Study the induction of apoptosis by naringenin, NARNPs, doxorubicin and doxorubicin combined with NARNPs in HepG2 and WI38 cells, the expression of apoptotic proteins, such as p53, and autophagy gene ATG5, LC3 gene were examined after treatment with naringenin, NARNPs, doxorubicin, and their combinations in HepG2 cells. Cultured HepG2 cells had very low level of p53 and after treatment with NARNPS. There are significantly increased in expression of P53 in NARNPs than Naringenin. This finding suggests that NARNPs are more effective at inducing p53 expression in HepG2 cells compared to naringenin. Increased p53 expression is typically associated with enhanced apoptosis, indicating that NARNPs may have a stronger apoptotic effect on HepG2 cells. This finding indicates that the treatment with IC50 concentrations of NARNPs and naringenin induces autophagy in HepG2 cells. This finding suggests that NARNPs are more effective at inducing autophagy in HepG2 cells than naringenin. This could indicate that NARNPs enhance the autophagic response, which might contribute to their overall cytotoxic effect. Additionally, From these studies, we observed that NARNPs decreased the cytotoxic effect of DOX in WI38 cell line (Fig. 5a, b, c).

## Discussion

The efficacy of chemotherapeutic drugs is often limited by various factors, including rapid clearance from the bloodstream, accumulation in unintended tissues and organs, high toxicity, enzymatic and hydrolytic breakdown and inefficient entry into cancer cells [53, 54]. In this study, the researchers focused on naringenin, a dietary phytochemical known for its pharmacological effects. Naringenin, a flavonoid found in citrus fruits, exhibits various anti-cancer, anti-inflammatory, antioxidant, and hepatoprotective properties through several modes of action. Its mechanisms include modulation of multiple signaling pathways, inhibition of cell proliferation, and induction of apoptosis. Naringenin causes cell cycle arrest in cancer cells by inhibiting cyclin and CDK. The increase in caspase, cytochrome c, and BAX and the downregulation of BCL2 in mitochondria are caused by naringenin, and the inhibition of ER-dependent mitogenic signaling cascades activation (e.g., phosphoinositide 3-kinase/AKT) or by induction of ER-dependent p38 kinase activation causes pro-apoptotic activities in cancer cells. Naringenin exhibits its angio-inhibitory effect by decreasing vascular endothelial growth factor (VGFG) and downregulating the TGF-pathway, thereby decreasing metastasis and invasion [55]. Naringenin induces the accumulation of p53, which subsequently leads to the expression of p21. The p21 protein then inhibits the cyclin E/cyclin-dependent kinase 2 (CDK2) complex, a key regulator of cell proliferation. The accumulation of p53 due to naringenin leads to cell cycle arrest. Apoptosis, a critical mechanism for eliminating cancer cells, is also induced by naringenin. This induction is marked by an increase in cytochrome c (CYPc) release and the elevation of pro-apoptotic proteins such as BCL2-associated X protein (Bax), BCL2-antagonist/killer 1 (Bak), and Caspase 3 (Cas3**)** [56]. At high doses, naringenin has demonstrated apoptotic and cytotoxic effects in several cancer cell lines, but it appears to have no detectable cytotoxic effects at lower concentrations. To address the issue of naringenin’s bioavailability, we developed NARNPs. The synthesized NARNPs had a size of approximately 50 nm and were investigated for their anticancer properties in HepG2 cells [57]. NARNPs are present in nanoparticulate formulations and the produced NARNPs were in the amorphous state [38]. Naringenin may inhibit the progression of cancer through a several of mechanisms, including: the mitochondrial damage, cell cycle arrest, the induction of apoptosis, different signaling pathways modification and ROS accumulation [58]. The study investigated the apoptotic and antiproliferative effects of naringenin, NARNPs, doxorubicin (DOX), and their combinations in HepG2 and WI38 cell lines. NARNPs, naringenin, DOX, and the combination of DOX with NARNPs demonstrated growth suppression in HepG2 cells, with respective IC50 values of 1.6 µg/ml, 22.32 µg/ml, and 0.46 µg/ml. NARNPs induced cell cycle arrest in the G0/G1 and G2/M phases and increased p53 expression, leading to apoptosis and cell cycle blockade [59]. NARNPs induce higher p53 expression and potentially stronger apoptotic effects in HepG2 cells compared to naringenin underscores the potential of NARNPs as a more effective therapeutic agent. NARNPs exhibit no cytotoxic effect on normal cells and reduce the cytotoxic effect of doxorubicin (DOX) when combined and suggests a promising strategy for enhancing cancer therapy selectivity. This selective targeting can improve the therapeutic index by maximizing cancer cell killing while minimizing harm to normal cells.

Phosphatidylserine is present in the inner leaflet of the plasma membrane. Phosphatidylserine turns to the outside of the cell membrane during the initial phases of apoptosis. Annexin-V-FITC can bind phosphatidylserine, which can then be utilized to identify apoptosis. A red fluorescence caused by propidium iodide in necrotic cells during the latter stages of apoptosis. Annexin V-FITC can enter the cytoplasm of necrotic cells due to the loss of membrane integrity. It then combines with the phosphatidylserine on the inner side of the membrane to produce green fluorescence [60]. The HepG2 cell dot pattern in the treatment group showed that its numbers of Annexin V-FITC positive and PI-negative cells, namely early apoptotic cells, significantly increased with the increase in concentration. Late apoptotic cells with Annexin V-FITC-stained double-positive cells. Natural products have a significant role in inducing apoptosis via the mitochondria-initiated death pathway in response to a variety of stimuli [61–63]. The mitochondria-mediated apoptosis pathway, crucial in many cancer therapies, is tightly regulated by the Bcl-2 protein family, which governs the balance between cell survival and death. Within this pathway, the proapoptotic gene Bax facilitates apoptosis by increasing mitochondrial membrane permeability and triggering the release of cytochrome c, while the antiapoptotic gene Bcl-2 counteracts this process to protect cells from apoptosis. A critical balance between these proteins determines the cell’s fate. Naringenin has been shown to influence this balance by increasing Bax expression and reducing Bcl-2 expression, tipping the scales towards apoptosis. Additionally, naringenin impacts the tumor suppressor gene p53 and its downstream target p21, both of which play pivotal roles in apoptosis. The activation of p53 leads to upregulation of Bax and suppression of Bcl-2, a process amplified by p21, which often works synergistically with p53 [64].

In Flow cytometry, the proportion of apoptotic cells is significantly increased by drug treatment, which is confirmed by apoptosis induction in HepG2 cells by NARNPs. The results demonstrate that NARNPs primarily induce apoptosis as a means of preventing HepG2 cell proliferation.

One fluorescent dye that is unique to double-stranded DNA is propidium iodide. Propidium iodide and double-stranded DNA combine to produce fluorescence, and the amount of double-stranded DNA present determines the fluorescence intensity. DNA fragments are created when apoptotic cells break their DNA. Due to the condensation of the DNA fragmentation of the apoptotic cells and nucleus, some of the DNA fragments were lost during the labeling procedure. Checkpoints that occur between the G1 and S phases and between the G2 and M phases control the cell cycle. These checkpoints are responsible for repairing damaged DNA or preventing an excessive progression of the cell cycle. The results of the experiment demonstrated that naringenin and NARNPs may damage cells’ DNA. Our findings indicate that NARNPs induced cell cycle arrest in the G0/G1 and G2/M phases. Cell cycle dysregulation and apoptosis induction are an important target in treatment of cancer. HepG2 cell cycle arrest was induced by NARNPs and naringenin at the Go/G1 and G2/M phases.

Induction of cancer cell apoptosis and cell cycle dysregulation is an important target in cancer therapy [65]. Treatment with naringenin elevated the protein expression levels of caspase-3 and caspase-9, which are critical mediators in the apoptotic pathway and play pivotal roles in the execution phase of apoptosis. Naringenin treatment upregulated the proapoptotic proteins Bax and Bak while simultaneously downregulating the antiapoptotic protein Bcl-2 [2]. Increasing ratio of Bax/Bcl-2, sequential activation of caspase-3, and release of cytochrome C in the mitochondrial-mediated apoptosis pathway are induced by naringenin [66, 67]. In previous reports, intracellular ROS generated by anticancer drugs killed their target cells. ROS is the second messenger in cell signaling that controls a variety of biological functions in both normal and tumor cells [68]. Cell cycle arrest in the G1 phase of the cell cycle is caused by P53 promoting transcription of the gene for the cyclin-dependent kinase inhibitory protein p21. Due to increased amounts of p21, cyclin E/cyclin-dependent kinase 2 and cyclin A/cyclin dependent kinase 2 kinases are inhibited, which prevents cell cycle progression [69–71]. Our results showed that the protein expression of p53 is increased by NARNPs. This finding further supports the potential of NARNPs as a therapeutic agent, given the role of p53 in promoting apoptosis and inhibiting tumor growth. Increased p53 expression can lead to enhanced activation of apoptotic pathways. The majority of chemotherapeutic drugs produce ROS, which is essential for causing cell death [72, 73]. Oxidative stress, cell death through apoptosis, and DNA damage caused by excessive ROS generation [74, 75]. Intercellular ROS generation by naringenin increased the suppression of cell growth [76, 77]. ROS plays a critical role in the processes that lead to cell death and autophagy [78].

To enable the breakdown of intracellular elements such as aggregated proteins, soluble proteins, macromolecular complexes, and foreign bodies, cells have to participate in the vital self-eating process known as autophagy [79]. Dysfunction of autophagy is associated with numerous human pathologies, including cancer and metabolic diseases such as diabetes, liver, heart, and lung diseases [80]. Autophagy can be induced by various exogenous stimuli, including mTOR inhibition, cytokines, hormones, hypoxia, nutritional deficiencies, and DNA damage. Drugs that target autophagy can act as antitumor drugs. Naringenin regulate the autophagy of cancer cells by targeting the mTOR signal [81]. Beclin-1 controls the use of ATG genes and protein products to activate the autophagy pathway. Naringenin was observed to increase the levels of microtubule-associated protein light chain 3 (LC3), along with the expression of ATG5 and Beclin 1, in a dose-dependent manner, underscoring its influence on the autophagic pathway [2]. Naringenin was demonstrated to regulate autophagy by decreasing the expression of p62, upregulating Beclin-1 expression, and increasing the LC3. These alterations indicate an enhancement in autophagic flux [82].

This study recommends conducting additional experiments to evaluate the effect of naringenin nanoparticles (NARNPs) on different types of cells, both cancerous and normal, such as breast, lung or pancreatic cancer cells. It is also recommended to measure the effect of these particles on a wider range of biochemical and molecular markers, such as other proteins related to apoptosis (Bcl-2, Bax) and oxidative stress factors, as well as inflammatory markers such as NF-κB and IL-6.

## Data Availability

No datasets were generated or analysed during the current study.

## Abbreviations

NARNPs: Naringenin nanoparticles
TEM: Transmission electron microscopy
FT-IR: Fourier transform infrared spectroscopy
XRD: X-ray diffraction
HCC: Hepatocellular carcinoma
PVA: Polyvinyl alcohol
MTT: 3-(4,5-dimethylthiazol-2yl)-2,5-diphenyl tetrazolium bromide
